# Complete mitochondrial genome of *Capreolus pygargus* (Cervidae: Capreolinae), a protected and threatened species in China

**DOI:** 10.1080/23802359.2019.1638842

**Published:** 2019-07-13

**Authors:** Tao Li, Yu-Kang Liang, Jian-Jun Zhang, Zhu-Mei Ren

**Affiliations:** a Institute of Applied Biology, Shanxi University, Taiyuan, China;; b School of Life Science, Shanxi University, Taiyuan, China;; c Shanxi Yangcheng Manghe Rhesus Monkey National Nature Reserve, Yangcheng, China

**Keywords:** *Capreolus pygargus*, Capreolinae, mitochondrial genome, phylogeny

## Abstract

We sequenced the complete mitochondrial genome (mitogenome) of Siberian Roe Deer, *Capreolus pygargus*, in China by the shotgun genome-skimming methods. The mitogenome of *C. pygargus* is totally 16,353 bp in length and contains 13 protein-coding genes (PCGs), 22 tRNA genes, two rRNA genes, and a control region. The sequence has a higher A + T content of 63.4% than G + C 36.6% and with a base composition of 33.4% A, 23.2% C, 13.4% G, and 30.0% T. All of the 13 PCGs initiate a typical ATN codon except *Nd4L* with GTG. Six PCGs terminate with a TAA codon, while *Cyt b, Atp8,* and *Nd1* terminate with AGA, TAG, and TA–, respectively. Whereas, *Cox3*, *Nd2*, *Nd3*, and *Nd4* terminate with a single T-. The phylogenetic trees of the subfamily Capreolinae with 13 PCGs indicated that *Capreolus* species were well-supported as a monophyletic group, which is sister to the clade of *Hydropotes* with well-support.

Siberian Roe Deer *Capreolus pygargus* is classified to the tribe Capreolini (Artiodactyla: Cervidae: Capreolinae), and was widely distributed in northeastern Asia, Siberia, Kyrgyzstan, Kazakhstan, and southern Ural Mountains (Bannikov 1954; Dulamtseren et al. 1989). As a widespread and common deer in its range, *C. pygargus* was listed in IUCN Red List of Threatened Species ver. 3.1 regarded as ‘Least Concern’ (Lovari et al. 2016). Matosiuk et al. (2014) reported the complete mitochondrial genome (mitogenome) of four *C. pygargus* individuals and analyzed the evolutionary neutrality of the genus *Capreolus* in Eurasia. So far, there was no study reported on the complete mitogenome of *C. pygargus* in China. Here, we sequenced the complete mitogenome of *C. pygargus* (GenBank Accession No. MK795818) in Shanxi, China, and constructed the phylogenetic relationship of *C. pygargus* and other Capreolinae species combined with the data from GenBank.

The muscle material was obtained from a dead adult individual of *Capreolus pygargus* that was killed by other animals and found by the forest ranger. The specimen was stored at the Manghe National Nature Reserve, Shanxi, China (Voucher No. Z14). We obtained the mitogenomic sequence of *C. pygargus* by the shotgun genome-skimming method on an Illumina HiSeq 4000 platform (Zimmer and Wen 2015). We performed the *de novo* assembly using SPAdes v. 3.7.1 (Bankevich et al. 2012) and annotated the complete mitogenome of *C. pygargus* within Geneious 11.0.3 using the complete mitogenomes of *Capreolus* species available from GenBank as references.

The complete mitogenome of *Capreolus pygargus* is a circular double-stranded DNA with 16,353 bp in length and contains 13 protein-coding genes (PCGs) (*Cox1*-*Cox3*, *Nd1*-*Nd6*, *Nd4L, Atp6*, *Atp8*, and *Cyt b*), 22 transfer RNA (tRNA) genes, two ribosomal RNA (rRNA) genes (*12S* and *16S rRNA*), and one control region (*D*-loop). The A + T content of 63.5% (33.5% A; 30.0% T) is much higher than that of G + C (36.5%) (23.2% C; 13.3% G). All the 37 genes are encoded on the H-strand except for eight tRNAs (*tRNA-Pro*, *Cys*, *tRNA-Ala*, *tRNA-Tyr*, *tRNA-Glu*, *tRNA-Ser*, *tRNA-Gln*, and *tRNA-Asn*), and one PCG (*Nd6*). The 13 PCGs initiate with ATN (ATA for *Nd2*, *Nd3* and *Nd5*, and ATG for *Cox1-Cox3*, *Atp6*, *Atp8*, *Nd1*, *Nd4*, *Nd6,* and *Cyt b*) except for *Nd4L* starting with GTG. Six of the 13 PCGs terminate with TAA (*Cox1*, *Cox2*, *Atp6*, *Nd4L*, *Nd5,* and *Nd6*), whereas *Cyt b* and *Atp8* terminate with AGA and TAG, respectively. Moreover, *Cox3* and *Nd1-Nd4* each terminated with a single T-. The genomic characters are similar to the other *Capreolus* spsecies (Matosiuk et al. 2014).

Based on the 13 PCGs, we constructed the phylogenetic tree using RAxML program under GTRGAMMA model and with 1000 bootstrap replicates (Stamatakis 2014). The phylogenetic relationship (Figure 1) supported the monophyly of the genus *Capreolus*, which is sister to the clade comprised by the genus *Hydropotes* within the same tribe Capreolini. However, two individuals from the species *C. capreolus* are grouped closely to these of the species *C. pygargus,* which we should further examine to confirm their classification by more samples and/or more data.

**Figure 1.**
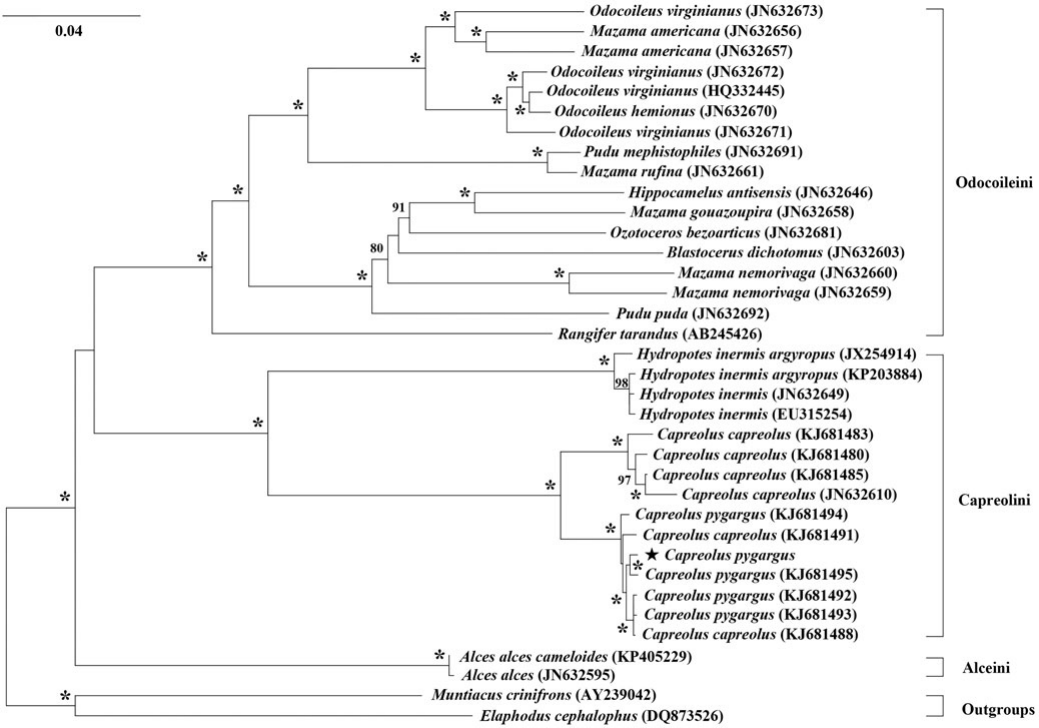
Phylogenetic tree of *Capreolus pygargus* and other Capreolinae species inferred from 13 protein-coding genes using maximum-likehood method with *Muntiacus crinifrons* and *Elaphodus cephalophus* as outgroups. Numbers associated with branches are ML-BS >70% and “*” represents nodes with 100% BS.
